# Human umbilical cord mesenchymal stem cell-derived extracellular vesicles promote lung adenocarcinoma growth by transferring miR-410

**DOI:** 10.1038/s41419-018-0323-5

**Published:** 2018-02-13

**Authors:** Liyang Dong, Yanan Pu, Lina Zhang, Qianqian Qi, Lei Xu, Wei Li, Chuan Wei, Xiaofan Wang, Sha Zhou, Jifeng Zhu, Xuefeng Wang, Feng Liu, Xiaojun Chen, Chuan Su

**Affiliations:** 1 Department of Pathogen Biology & Immunology, State Key Lab of Reproductive Medicine, Jiangsu Key Laboratory of Pathogen Biology, Nanjing Medical University, Jiangsu 211166 Nanjing, P. R. China; 2 Central Laboratory, The Affiliated Hospital of Jiangsu University, Jiangsu 212002 Zhenjiang, P. R. China

## Abstract

Although accumulating evidence has linked mesenchymal stem cells (MSCs) with tumor growth, the underlying mechanisms are poorly understood. Here, we demonstrated for the first time that human umbilical cord MSCs (hUCMSCs) dramatically increased the growth of lung adenocarcinoma (LUAD) cancer cells in a xenograft tumor model. Then, we observed that hUCMSC-derived extracellular vesicles (hUCMSC-EVs) contribute to the hUCMSC-promoted LUAD cell growth through a direct effect on LUAD cells. Furthermore, we showed that hUCMSC-EV-mediated LUAD growth is associated with increased proliferation and decreased apoptosis in LUAD cells, concomitant with reduced PTEN expression mediated by the hUCMSC-EV-transmitted miR-410. Our findings provide novel insights into the intercellular communications between cancer cells and MSCs through MSC-EV-miRNA and suggest that modification of hUCMSC-EVs might be an attractive therapeutic option for the clinical application of hUCMSC-EVs that would reduce unwanted side effects.

## Introduction

Mesenchymal stem cells (MSCs) are multipotent cells that reside in various tissues and have the potentials to differentiate into mesenchymal cells, including osteoblasts, adipocytes, and chondrocytes^1^. MSCs can be recruited to sites of inflammation and injury, where they contribute to the tissue regeneration following damage^2^, suggesting that MSCs have considerable therapeutic potentials in tissue regeneration^3^. Meanwhile, numerous studies have confirmed that MSCs can also migrate into the tumor microenvironment^4^, which has led to increased interest in using MSCs as carriers to deliver anti-tumor drugs or genes for cancer treatment^5^.

Bone marrow-derived MSCs (BM-MSCs) are the most common cell source, especially in animal-based experiments, for tissue repair, engineering, and vehicles for cell-based gene therapy. However, the clinical application of BM-MSCs is limited due to the invasive nature of the sample collection, low cell yield, reduced proliferation, and differentiation capacities in aging donors^6^, and some existing ethical concerns. Unlike BM-MSCs, human umbilical cord-derived MSCs (hUCMSCs) are viewed as a better choice of MSCs for clinical application due to the painless collection procedure, high cell vitality, low immunogenicity, high paracrine potential for accelerating injury tissue repair processes, and the absence of ethical issues^7,8^. Moreover, banks of hUCMSCs are being set up in many countries^9^.

However, accumulating evidence has shown that MSCs participate in the formation of the cancer microenvironment and the promotion of tumor growth^10,11^. In addition to direct trans-differentiation effects toward cancer-associated fibroblasts and immunosuppressive effects^12,13^, MSCs can also promote tumor growth through numerous bioactive factors^14^. However, the exact mechanisms that underlie the promotion of tumorigenesis by MSCs have remained obscure. Given the high incidence of cancer, including lung cancer, gastric cancer, and breast cancer, and the fact that early diagnosis for cancer is difficult, the risk of oncogenicity has cast a shadow over future clinical application of MSCs. Among the cancers of concern, lung cancer is one of the most malignant tumors and a leading cause of cancer-related mortality. Specifically, lung adenocarcinoma (LUAD) accounts for ~50% of all lung cancers^15^. Although several studies have revealed the relationships between MSCs derived from bone marrow and LUAD growth^16,17^, the roles of MSCs from human umbilical cord in LUAD progression have not been exhaustively investigated. Thus, exploring the effects and underlying mechanisms of hUCMSCs on LUAD growth will be the key for assuring maximal safety of future clinical application of hUCMSCs.

In this study, we found that hUCMSCs significantly promoted LUAD growth. Further experiments confirmed that hUCMSC-derived extracellular vesicles (hUCMSC-EVs) contributed to the hUCMSC-promoted LUAD cell growth, which was associated with the translocation of miR-410 to LUAD cells that directly inhibited the expression of PTEN. Our findings provide new insights indicating that the tumor promotion by hUCMSCs is through MSC-EV-miRNA and suggest that manipulation of hUCMSC-EVs might be a therapeutic option to potentially reduce the side effects in future clinical application of hUCMSCs.

## Results

### The hUCMSCs and their EVs promoted LUAD cell growth in vivo

**Fig. 1:**
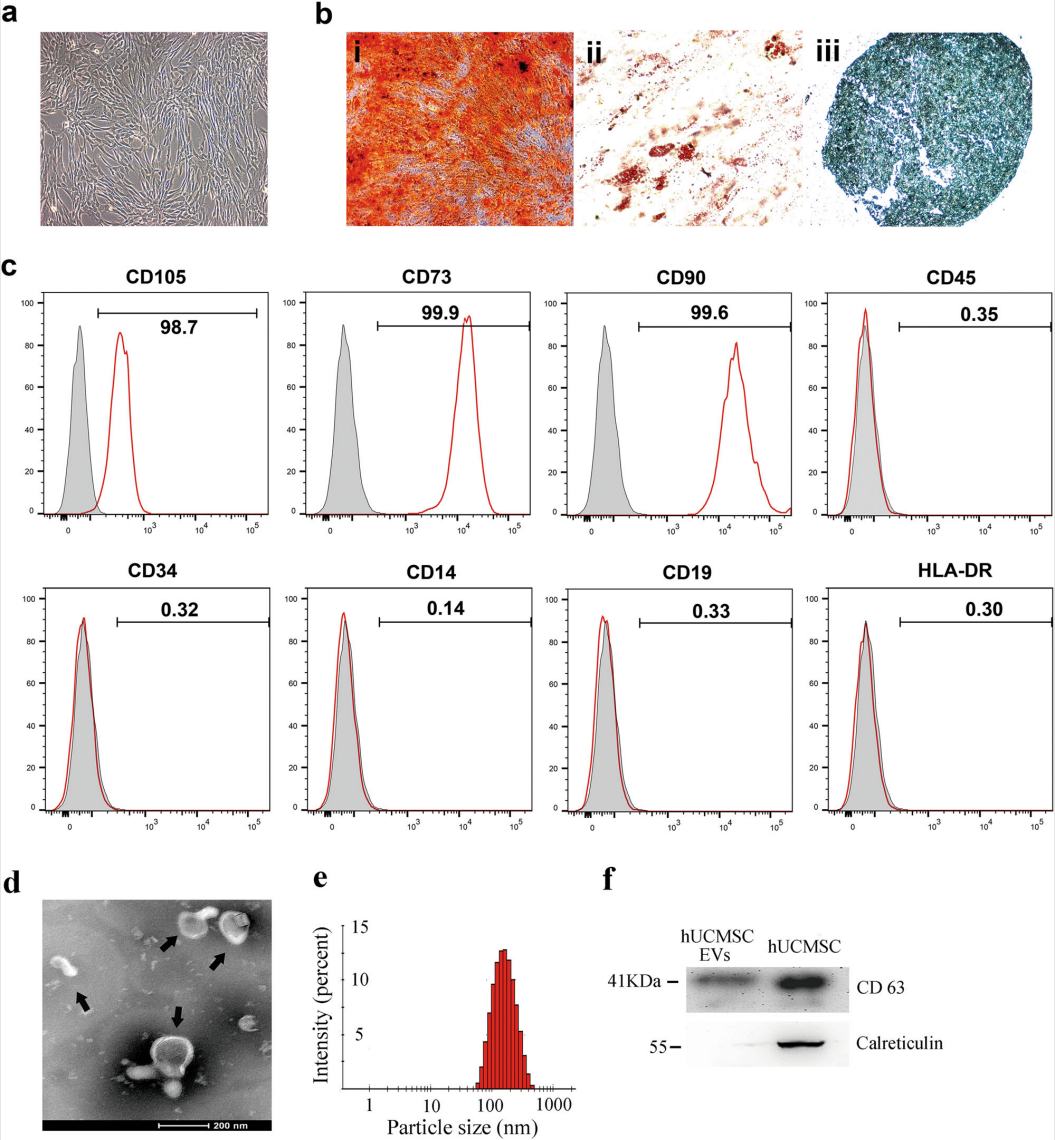
Identification of human umbilical cord mesenchymal stem cells (hUCMSCs) and hUCMSC-derived extracellular vesicles (hUCMSC-EVs). **a** The cell morphology of hUCMSCs (passage 3) was observed under a light microscope (magnification, ×100). **b** Representative images of osteocyte (×100), adipocyte (×400), and chondrocyte (×200) differentiation of hUCMSCs cultured in the differentiation media. The cells were analyzed using cytochemical staining with Alizarin Red (**i**), Oil red O (**ii**), and Alcian Blue (**iii**), respectively. **c** Flow cytometric analysis of the expression of cell surface markers related to MSCs. **d** Transmission electron microscopic images of hUCMSC-EVs. The scale bars indicate 200 nm, and the arrows indicate typical hUCMSC-EVs. **e** The size distribution of the hUCMSC-EVs was examined using a Zetasizer Nano ZS. **f** The positive marker for EVs, CD63, was detected in hUCMSC-EVs using western blotting, whereas the negative marker calreticulin was not detected

**Fig. 2:**
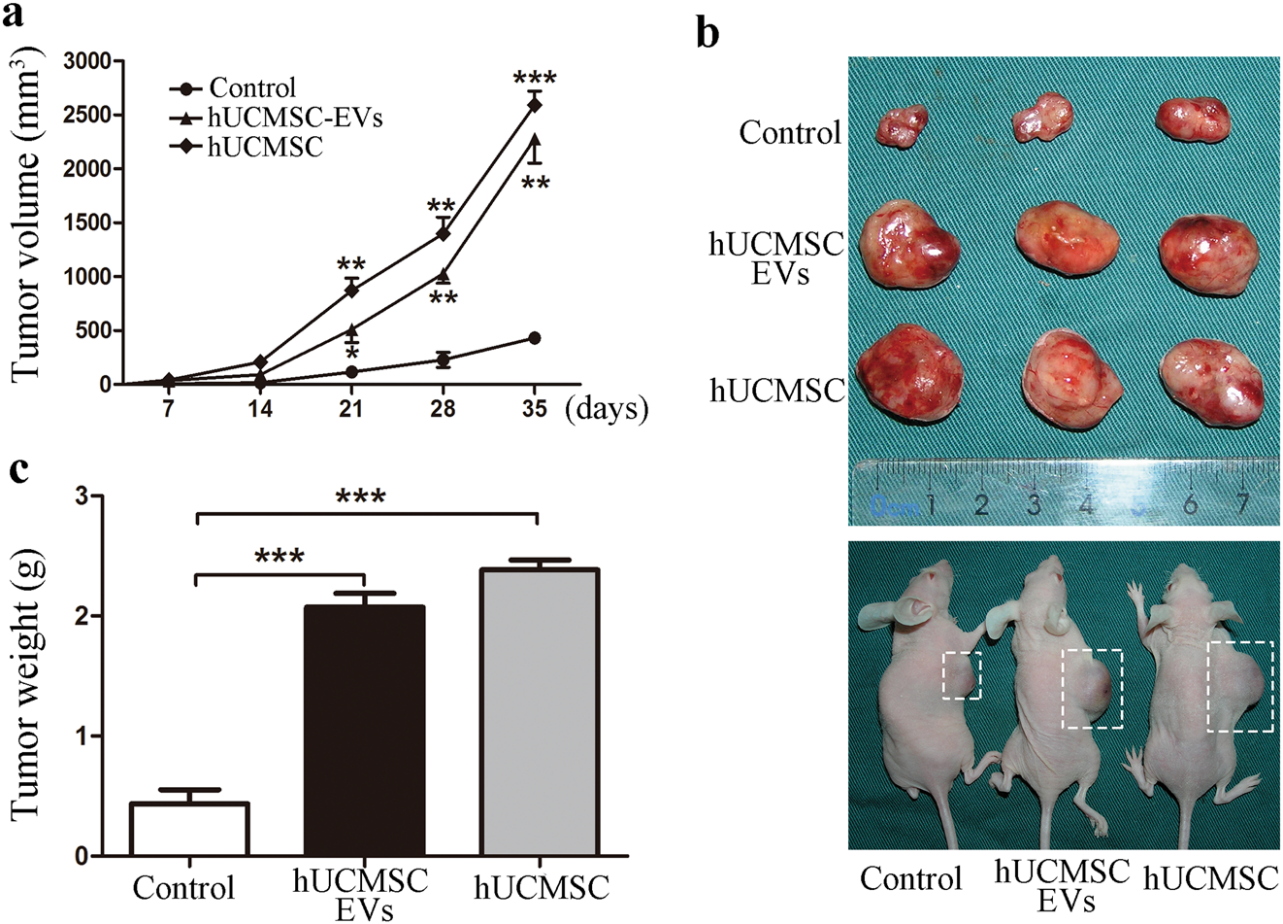
HUCMSCs and hUCMSC-EVs facilitate the growth of inoculated tumors. **a** The tumor volumes in the mice from the groups implanted with H1299 cells alone or H1299 cells co-implanted with hUCMSCs or hUCMSC-EVs. **b** Measurements of the tumor weights of the H1299 xenograft tumors at 35 days post implantation. **c** Imaging of the tumors and tumor-bearing mice from the groups implanted with H1299 cells alone or H1299 cells co-implanted with hUCMSC-EVs or hUCMSCs at 35 days post implantation

The hUCMSCs were purified (Fig. 1a–c) and confirmed on the basis of the criteria defined by International Society for Cellular Therapy^18^. To evaluate the effects of hUCMSCs on LUAD growth, we established a xenograft model in which H1299 cells or PC-9 cells were mixed with hUCMSCs and subcutaneously injected into nude mice. The tumor growth under the influence of hUCMSCs was faster than that in the vehicle control group, as indicated by the measurements of the tumor sizes (Fig. 2 and Supplementary Figure S1). However, there was no tumor formation in the hUMSCs-only group. These results suggest that the hUCMSCs promote LUAD cell growth.

EVs, a paracrine factor secreted by MSCs, play a very important role in the actions of MSCs^14,19^. We investigated whether EVs were responsible for the pro-growth activity of hUCMSCs. The hUCMSC-EVs were isolated and purified from the hUCMSC-conditioned medium. As shown in Fig. 1d–f, the hUCMSC-EVs were typical round-shaped membrane particles with sizes that ranged from 60 to 450 nm in diameter with an average of 142 nm and expressed CD63 (a representative marker of EVs), whereas no calreticulin (an intracellular contaminant) was present. Interestingly, further results showed that tumor growth in the hUCMSC-EV co-implantation mice has the similar tendency to that in hUCMSC co-implantation mice. Indeed, the tumor growth in hUCMSC-EV or hUCMSC co-implantation mice was significantly faster than that in mice implanted with the H1299 cells alone (Fig. 2). Taken together, these results suggest that hUCMSC-EVs contribute to the hUCMSC-promoted LUAD cell growth.

### The hUCMSC-EVs promoted LUAD growth in vivo through a direct effect on LUAD cells

**Fig. 3:**
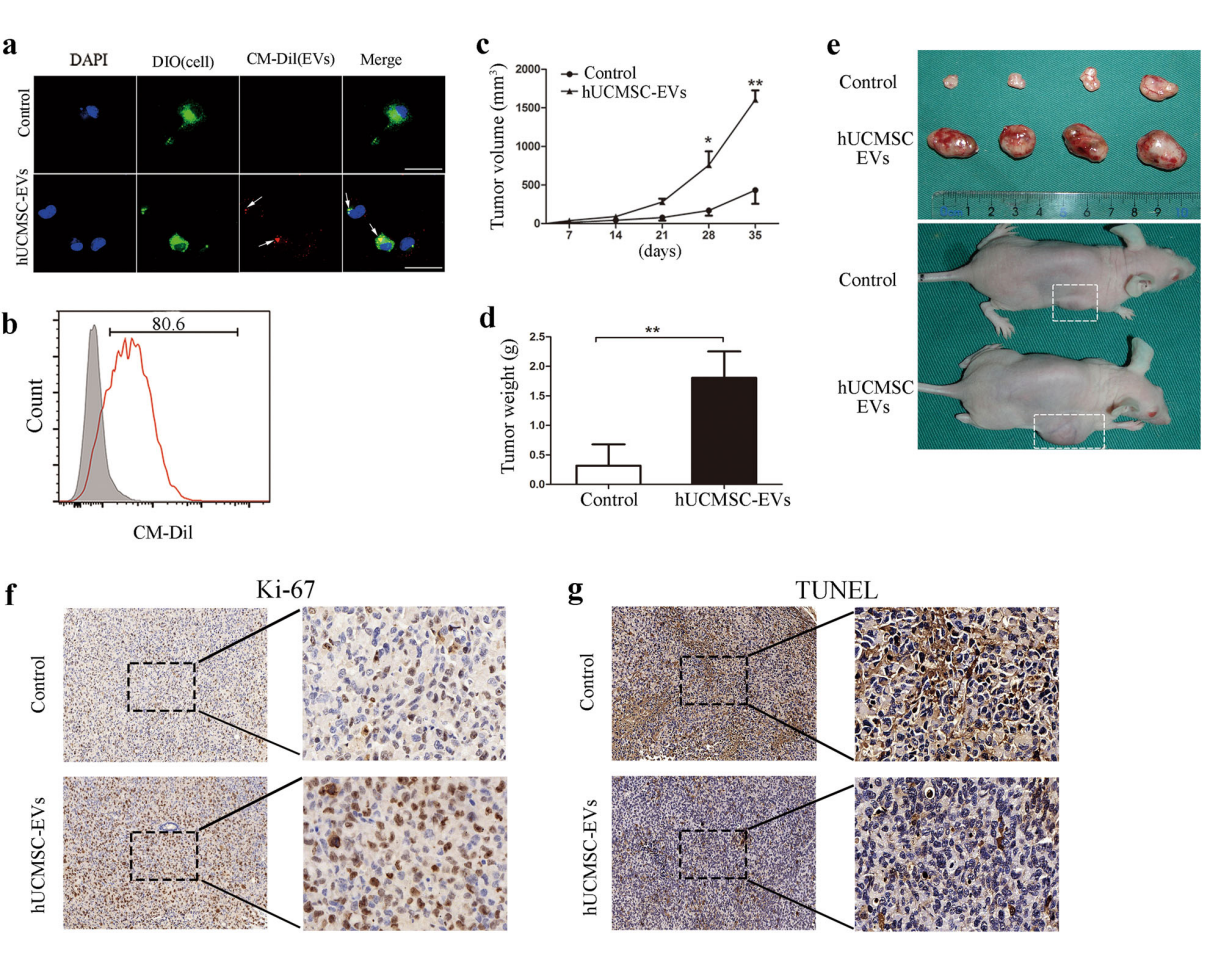
Effects of hUCMSC-EVs pre-stimulation on tumor growth in vivo. **a** DIO-labeled H1299 cells (green) were incubated with CM-Dil-labeled hUCMSC-EVs (red) for 2 h, and the hUCMSC-EV uptake by the LUAD cells was determined. The arrows indicate the fusion of the membrane. The scale bars indicate 50 μm. **b** FACS analysis of H1299 cells that had been treated for 12 h with CM-Dil-labeled hUCMSC-EVs or serum-free medium as control. The H1299 cells were pre-stimulated with hUCMSC-EVs for 12 h before they were injected into the nude mice. **c** The tumor volumes were measured, and **d** the tumor weights were determined at 35 days. **e** Imaging of tumors and tumor-bearing mice from the hUCMSC-EV-pre-stimulated groups at 35 days after the injections. **f** Ki-67 and **g** TUNEL immunohistochemical staining on tumor sections from the control or hUCMSC-EV-pre-stimulated group. The images are shown at ×100 (left panel) and ×400 (right panel)

MSC-EVs might promote LUAD growth in vivo through either a direct effect on LUAD cells or an indirect effect via other cells, such as endothelial cells and immune cells in the xenograft model in which LUAD cells were mixed with hUCMSC-EVs. To investigate whether the hUCMSC-EV-promoted LUAD growth was associated with a direct alteration of the physiological state of LUAD cells, we first pre-treated LUAD cells with CM-Dil-labeled hUCMSC-EVs in vitro and observed that hUCMSC-EVs could fuse with the membranes of the LUAD cells (Fig. 3a). Moreover, ~80% of the total LUAD cells that were treated with the CM-Dil-labeled hUCMSC-EVs were CM-Dil-positive (Fig. 3b), which suggested that the hUCMSC-EVs can be taken up by LUAD cells. Next, we injected pre-stimulated LUAD cells subcutaneously into BALB/c nude mice. Interestingly, we found that the hUCMSC-EV pre-treatment enhanced the tumor progression and growth in vivo (Fig. 3c–e and Supplementary Figure S2). These in vivo findings demonstrate that the hUCMSC-EVs promote tumor growth through a direct effect on LUAD cells.

In addition, more Ki-67-positive and fewer terminal transferase-mediated dUTP nick-end labeling (TUNEL)-positive cells were observed in the tumor sections from the hUCMSC-EV pre-treated group (Fig. 3f, g). This result indicates that increased proliferation and decreased apoptosis of lung cancer cells may contribute to the hUCMSC-EV-mediated LUAD growth.

### The hUCMSC-EVs enhanced LUAD cell growth in vitro

**Fig. 4:**
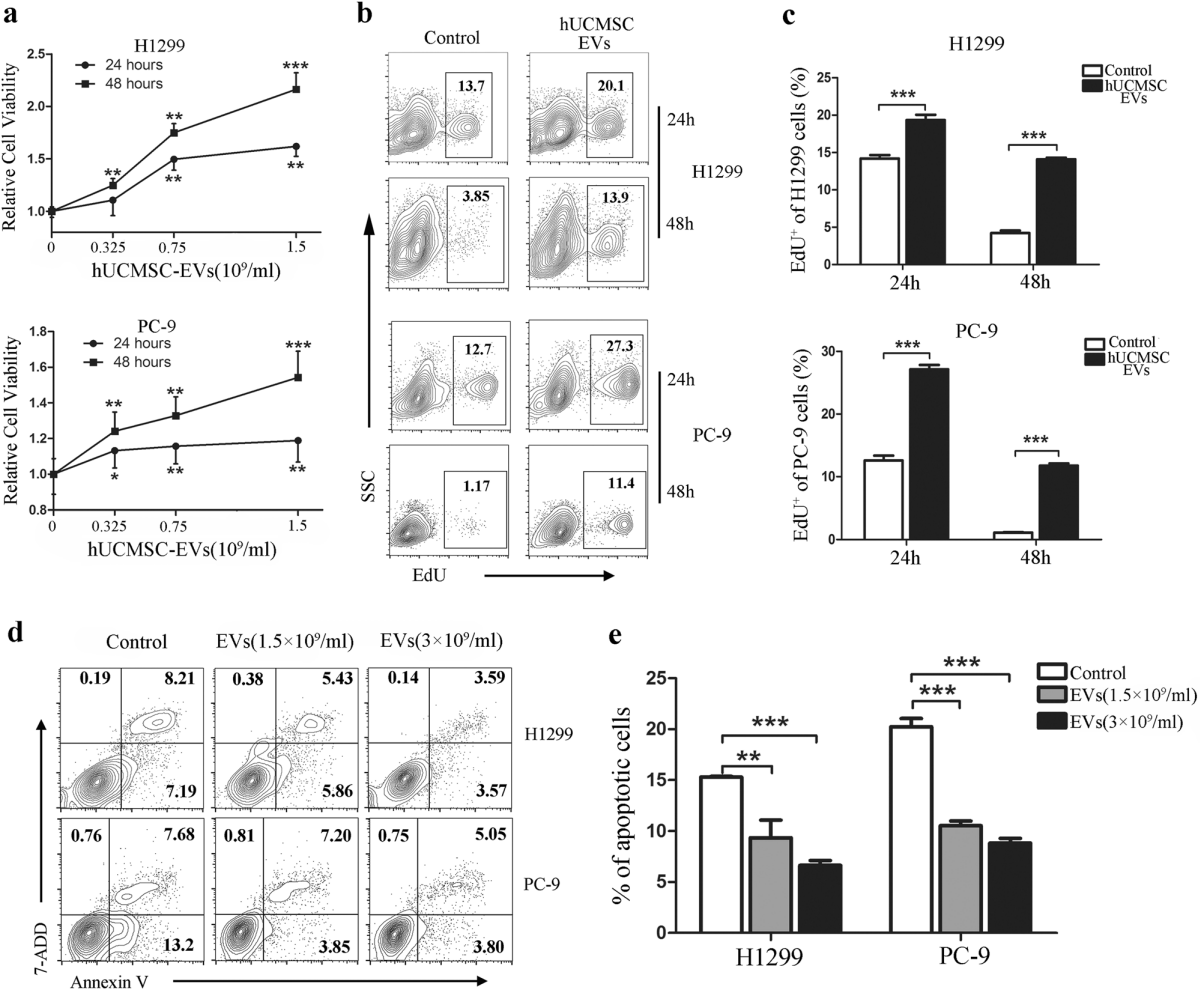
HUCMSC-EVs increase the viability and proliferation of the LUAD cells and decrease their apoptosis. **a** H1299 or PC-9 cells in serum-free media were incubated with the indicated amounts of hUCMSC-EVs for 24 or 48 h, and the cell viability was determined using the CCK8 assay. **b** H1299 or PC-9 cells in serum-free medium were treated with 1.5 × 10^9^ per mL of hUCMSC-EVs for 24 or 48 h, and the cell proliferation was measured using an EdU cell proliferation assay. Representative contour plots of the EdU-positive cells are shown. **c** The percentages of EdU-positive cancer cells are shown. **d** H1299 or PC-9 cells were cultured in serum-free conditions and treated with various amounts of hUCMSC-EVs for 24 (H1299 cells) or 48 h (PC-9 cells). The apoptotic cells were determined by staining with 7-aminoactinomycin D (7-AAD) and Annexin-V after the treatment. Representative contour plots of the cell apoptosis are shown. The proportions of dead cells (Annexin V^−^/7-ADD^+^), live cells (Annexin V^−^/7-ADD^−^), early apoptotic cells (Annexin V^+^/7-ADD^−^), and late apoptotic/necrotic cells (Annexin V^+^/7-ADD^+^) were compared. **e** The percentages are shown for both early and late apoptotic cells. The data are expressed as the means ± SD of at least three replicates and *P < 0.05; **P < 0.01; ***P < 0.001, versus control (one-way ANOVA)

Our in vitro results showed that the hUCMSC-EVs significantly increased the viability of LUAD cells in a time- and dose-dependent manner (Fig. 4a). The proliferation of the LUAD cells was also remarkably increased (Fig. 4b, c). In addition, our results indicated that the hUCMSC-EVs dramatically reduced LUAD cell apoptosis in a dose-dependent manner (Fig. 4d, e). These findings correlated well with the observations that there were more Ki-67-, but few TUNEL-positive cells in the tumor sections from the mice that were injected with LUAD cells pre-treated with hUCMSC-EVs.

### The hUCMSC-EVs promoted LUAD cell growth by transferring miR-410

**Fig. 5:**
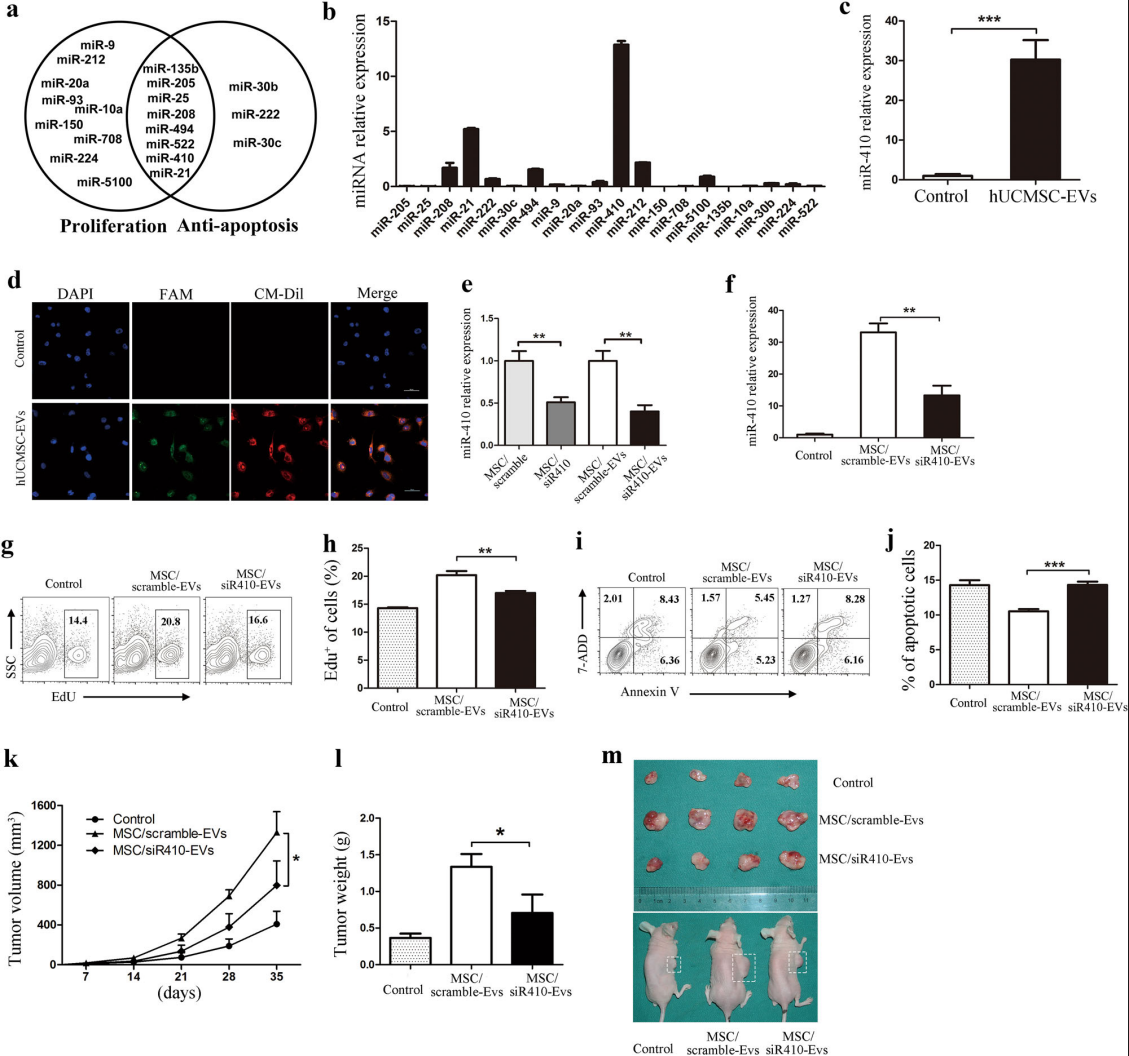
EVs from hUCMSCs promote LUAD cell growth partly by transferring miR-410. **a** The predominant miRNAs that have been reported to participate in LUAD cell proliferation and anti-apoptosis. **b** Real-time PCR analysis of the predominant miRNAs reported to be present in hUCMSC-EVs. The expression of the miRNA in hUCMSC-EVs was normalized to the U48 levels. **c** The expression of miR-410 in H1299 (24 h) cells that were treated with hUCMSC-EVs (1.5 × 10^9^ per mL). **d** EVs from hUCMSCs that had been transfected with a FAM-miR-410 mimic (green) were labeled with CM-Dil (red) and added to H1299 cells for 6 h, then evaluated using laser confocal microscopy. Scale bars: 50 μm. **e** The levels of miR-410 in the MSC/siR410 and MSC/siR410-EVs. **f** The miR-410 expression in H1299 cells treated with MSC/siR410-EVs. **g**–**j** H1299 cells were incubated with MSC/scramble-EVs or MSC/siR410-EVs (1.5 × 10^9^ per mL) for 24 h. **g** Representative flow cytometric contour plots of the EdU-stained H1299 cells as indicators of the proliferation are shown. **h** The percentages of EdU-positive cancer cells are shown. **i** Representative contour plots of the Annexin V/7-ADD dual-stained H1299 cells as an indicator of apoptosis are shown. **j** The percentages of both early and late apoptotic cells are shown. **k**–**m** The H1299 cells (1.5 × 10^6^) were pre-stimulated with MSC/scramble-EVs or MSC/siR410-EVs for 12 h before they were injected into the nude mice. **k** The tumor volumes were measured, and **l** the tumor weights were determined at 35 days. **m** Imaging of tumors and tumor-bearing mice from the control, MSC/scramble-EVs, and MSC/siR410-EVs groups at 35 days after the injections. The data are expressed as the means ± SD and *P < 0.05; **P < 0.01; ***P < 0.001

Studies have shown that EVs play a pivotal role in cellular communication by transferring their contents, especially miRNAs^20,21^, and accumulating evidence indicates that miRNAs are considered to be key mediators of LUAD cell growth^22^. We therefore hypothesized that hUCMSC-EVs may affect LUAD cell growth by delivering miRNAs. We detected 20 miRNAs (Fig. 5a) that have been widely reported to be LUAD growth-supportive miRNAs^22–39^. In particular, we found a strong enrichment of miR-410 in hUCMSC-EVs (Fig. 5b). Furthermore, the expression of miR-410 in hUCMSCs was higher than that in LUAD cells (Supplementary Figure S3). More importantly, the level of miR-410 was significantly increased in the LUAD cells after treatment with hUCMSC-EVs (Fig. 5c and Supplementary Figure S4e). In addition, the green fluorescence and the red fluorescence were co-located in the cytoplasm of LUAD cells that had been treated with CM-Dil-labeled hUCMSC-EVs (red) containing miR-410 (FAM, green), suggesting that miR-410 can be transferred into LUAD cells through hUCMSC-EVs (Fig. 5d).

To further confirm that hUCMSC-EVs may promote the growth of LUAD cells by transferring miR-410, we performed miR-410 knockdown in hUCMSCs using a miR-410 inhibitor, which led to a decrease in the miR-410 expression in the hUCMSC-EVs (MSC/siR410-EVs) (Fig. 5e). As expected, the level of miR-410 was markedly lower in hUMSC/siR410-EV-treated cells than that in control cells (Fig. 5f), indicating that miR-410 in hUCMSC-EVs contributed to the increased miR-410 expression in hUCMSC-EV-treated cells. Furthermore, hUMSC/siR410-EVs induced significantly less proliferation, but more apoptosis in the H1299 cells (Fig. 5g–j). More importantly, compared with MSC/scramble-EVs group, MSC/siR410-EVs significantly inhibited LUAD cell growth in nude mice (Fig. 5k–m and supplementary Figure S2). As expected, miR-410 was also a major contributor to hUCMSC-mediated LUAD cells growth in vivo (Supplementary Figure S1). Taken together, these results suggest that transferring miR-410 is one of the mechanisms by hUCMSC-EVs to promote LUAD cell growth.

### PTEN was involved in the miR-410-mediated LUAD cell growth

**Fig. 6:**
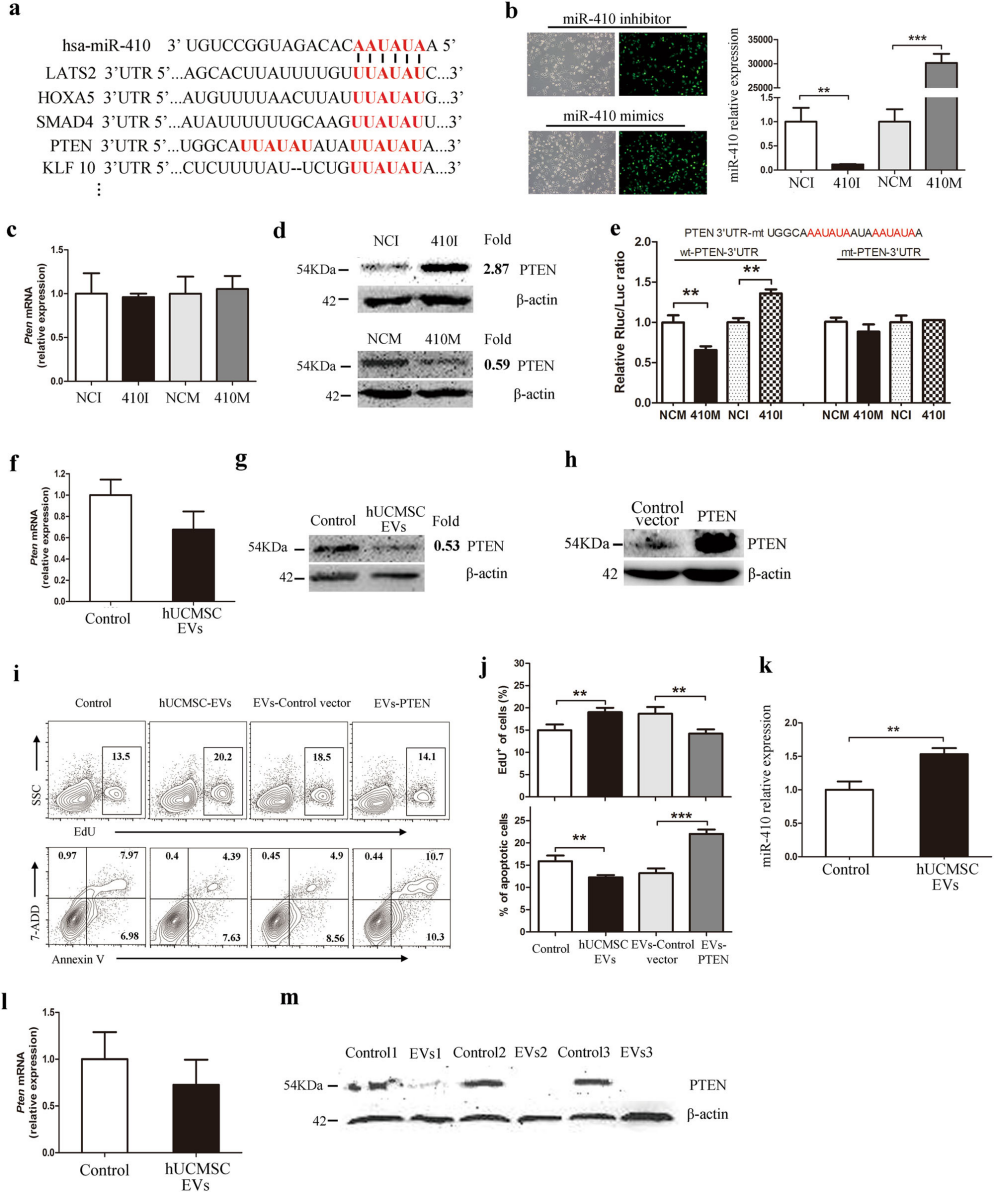
PTEN may be involved in the miR-410-mediated LUAD cell growth. **a** Schematic diagram of the putative miR-410-binding site in the 3′UTR regions (red) of LATS2, HOXA5, Smad4, PTEN, and KLF 10. **b** The transfection efficiency of H1299 cells was estimated by fluorescence microscopy 6 h (×100) after transfection of the FAM-miR-410 inhibitor and FAM-miR-410 mimics (left panel). The results are presented as light microscopic images of the FAM-expressing cells. Real-time PCR was used to investigate the miR-410 transfection efficiency (right panel). **c** Real-time PCR analysis of the expression of PTEN in H1299 cells at 24 h post transfection of the miR-410 inhibitor or miR-410 mimics compared to their negative controls. **d** PTEN protein expression was analyzed by western blotting at 48 h post transfection with β-actin as a loading control. The intensity of each band was analyzed using ImageJ software, and the ratios of the miR-410 inhibitor to the inhibitor control and of the miR-410 mimics to the mimic control are presented as the fold differences. **e** MiR-410 dramatically suppressed the luciferase activity that carried wild-type but not mutant 3′UTR of PTEN. **f**,** g** H1299 cells were treated with hUCMSC-EVs (1.5 × 10^9^ per mL) for 24 h or 48 h. The expression of PTEN mRNA **f** and protein **g** was determined by real-time PCR or western blotting, respectively. **h** H1299 cells were transfected with control vector plasmid or PTEN overexpression plasmid and the PTEN protein expression were analyzed by western blot. **i**,** j** H1299 cells, which were transfected with PTEN overexpression plasmid, were incubated with/without hUCMSC-EVs (1.5 × 10^9^ per mL) for 24 h. **i** Representative flow cytometric contour plots of the EdU-stained H1299 cells and the Annexin V/7-ADD dual-stained H1299 cells were shown. **j** The percentages of EdU-positive cancer cells and both early and late apoptotic cells were shown. **k** MiR-410 expression, **l** PETN mRNA expression, and **m** PETN protein expression in the tumors of the control and hUCMSC-EV-pre-stimulated H1299 cell groups of nude mice. The data are expressed as the means ± SD of three replicates and **P < 0.01; ***P < 0.001 (Student’s *t* test)

To identify the targets of the miR-410 that could contribute to the enhanced LUAD cell growth, we predicted potential targets of miR-410 by in silico analysis using miRanda and TargetScan (www.microrna.org and www.targetscan.org). The predicted candidates included serine/threonine-protein kinase 2 (LATS2), homeo box A5 (Hoxa5), Smad4, phosphatase and tensin homolog deleted on chromosome ten (PTEN), Krüppel-like factor 10 (KLF 10) et al (Fig. 6a). Among the candidates, PTEN was selected for further analysis, owing to its relatively high prediction score and its two complementary structures with miR-410 (Fig. 6a). Moreover, PTEN has been proven to have a major impact on the proliferation and apoptosis of LUAD cells^40,41^. We transfected LUAD cells with the miR-410 inhibitor or miR-410 mimics, and found that the PTEN protein expression but not the *PTEN* messenger RNA (mRNA) was dramatically increased or decreased in miR-410 inhibitor- or miR-410 mimic-transfected LUAD cells, respectively (Fig. 6b–d and Supplementary Figure S4a–c), suggesting that miR-410 can regulate PTEN expression at the post-transcriptional level in LUAD cells. To further identify whether miR-410 directly binds the 3′UTR region of PTEN, we conducted chimeric constructs, which harbor luciferase mutant 3′UTR sequence (mt-PTEN-3′UTR) or wild-type 3′UTR sequence (wt-PTEN-3′UTR). As shown in Fig. 6e and Supplementary Figure S4d, overexpression of miR-410 repressed the luciferase activity of the reporter gene within the wild-type construct but not the mutant PTEN 3′UTR construct, while miR-410 inhibition increased the luciferase activity. These results suggest that miR-410 directly targets PTEN in LUAD cells.

Interestingly, we found that PTEN protein expression, but not *PTEN* mRNA, was significantly decreased in LUAD cells that were exposed to hUCMSC-EVs (Fig. 6f, g and Supplementary Figure S4f, g). In order to identify the role of PTEN in the hUCMSC-EV-mediated LUAD cell growth, we constructed an overexpression PTEN gene plasmid (PTEN) and control vector plasmid (control vector), and transfected these plasmids into LUAD cells before treating these cells with or without hUCMSC-EVs. The upregulation efficiency of PTEN plasmid was detected by western blotting (Fig. 6h and Supplementary Figure S3h). Strikingly, overexpression of PTEN abolished the promotion of LUAD cell proliferation by hUCMSC-EVs, while restoring LUAD cell apoptosis (Fig. 6i, j and Supplementary Figure 4i, j), suggesting that PTEN was involved in hUCMSC-EV-mediated LUAD cell growth. More importantly, the expression of PTEN protein was also decreased in the tumor tissues in the hUCMSC-EV-pre-treated mice, which paralleled the elevated expression of miR-410 in the tumor tissues (Fig. 6k–m). Taken together, these results suggest that hUCMSC-EVs reduce PTEN expression by transferring miR-410 and that this process is involved in the hUCMSC-EV-mediated LUAD cell growth.

## Discussion

The ability of MSCs to localize to the sites of tumors has inspired the investigation of these cells as potential anti-tumor tools for the transport of anti-tumor genes or drugs^5,42^. However, there is accumulating evidence that MSCs participate in the formation of the cancer microenvironment and promote tumor growth^10,11^. In addition, with the currently available techniques, the early formation of many types of tumors, including lung cancer, cannot be detected, which tremendously limits the clinical application of MSCs. Compared to other fetal and adult tissue-derived MSCs, hUCMSCs are easier to isolate and expand. Moreover, hUCMSCs have low immunogenicity, which enhances their great potential for clinical application^7,8^. However, the effect of hUCMSCs on tumor growth has not been exhaustively studied, especially in lung cancer, which is a leading cause of cancer-related deaths throughout the world. Recently, a co-injection study in nude mice revealed that human Wharton’s jelly MSCs (hWJMSC) can increase the growth of the LUAD-derived cancer stem cell lines AC-229 and AC-223^43^. However, the mechanism is unknown. In this study, we found that hUCMSCs have the ability to promote the growth of the LUAD cells in nude mice.

In recent years, most reports have concluded that secreted paracrine factors are critical mediators of the role of MSCs in tumor progression^14,19^. EVs, a type of MSC paracrine factor, are small membranous vesicles that can mediate cell-to-cell communication by transferring biological material to adjacent or distant cells^20^. In this study, we showed that hUCMSC and their EVs enhanced LUAD cell growth by increasing the proliferation of LUAD cells and/or reducing their apoptosis. Our results further support the concept that MSC-EVs contribute to MSC-mediated tumor progression^44^. However, contradictory results regarding MSC-EV roles in tumor growth have been described^45^, perhaps mainly because of differences in MSC source, tumor types, and animal model and so on. Recently, studies in regenerative medicine have shown that MSC-EVs are able to mimic the therapeutic effects of the MSCs in kidney^46^, cardiac^47^, and various lung injury diseases^48,49^. These results suggest that MSC-EVs might be novel and better therapeutic tools than MSCs^50^. However, the data obtained in this study raised concerns regarding the use of hUCMSC-EVs as therapeutic interventions for humans in the future and suggested that caution is required for their use under malignant conditions, at least for LUAD.

Various studies have shown that many miRNAs are involved in the growth of LUAD cells^22^. In addition, many miRNAs have been shown to be selectively packaged into EVs, and several studies have confirmed that EV-transferred miRNA can modulate the activities of target cells^20,21^. However, whether hUCMSC-EVs can affect tumor cell growth by transferring miRNAs is unknown. In present study, we first demonstrated that many miRNAs in hUCMSC-EVs are either involved in the proliferation or anti-apoptotic effects in LUAD cells. Based on our results that the expression of miR-410 was significantly increased in the LUAD cells treated with hUCMSC-EVs and that miR-410 can be transferred into LUAD cells through hUCMSC-EVs, we hypothesized that hUCMSC-EVs may promote LUAD cell growth through the transfer of miR-410. Although we found that the level of miR-410 was significantly decreased in the LUAD cell after treatment with MSC-siR410-EVs, our study still could not rule out that hUCMSC-EVs can also induce the endogenous expression of miR-410 in LUAD cell. Indeed, we observed that hUCMSC/siR410-EVs just partially impaired the growth-promoting effects of hUCMSC-EVs on LUAD, which supported our hypothesis. However, our present results could not exclude the possibility that other substances in hUCMSC-EVs, such as other miRNAs, some proteins or lipids, may also play roles to support LUAD growth. Indeed, several studies reveal that MSC-EVs could also promote tumor growth by inducing angiogenesis, activating Akt and ERK1/2 signaling pathways^51^. Our findings suggest that engineered EVs might be a novel treatment strategy for the clinical application of MSC-EVs. More evidence is needed to address this concern.

MiRNA can regulate target gene expression by inducing mRNA degradation or translational inhibition. Using computational bioinformatics, we predicted that PTEN was one of potential targets of miR-410. PTEN, a tumor suppressor gene, has been proven to impact the proliferation and apoptosis of LUAD cells^40,41^. In the present study, we confirmed that PTEN is a direct target of miR-410 and that miR-410 regulates PTEN expression through post-transcriptional level. Consistent with this concept, the protein but not the mRNA expression of PTEN was decreased in the LUAD cells treated with hUCMSC-EVs. We further examined the mRNA and protein expression of PTEN in hUCMSC-EVs using real-time PCR and western blotting, respectively, in which they were not detectable (Supplementary Figure 5), suggesting that hUCMSC-EVs probably do not transfer the PTEN mRNA or protein to the LUAD cells but instead act through an indirect induction. These results suggest that hUCMSC-EVs could transfer miR-410 to the LUAD cells and subsequently inhibit the PTEN protein expression, thus affecting the LUAD cell growth.

In conclusion, our study demonstrates that hUCMSC-EVs contribute to hUCMSC-mediated LUAD growth, and this effect may be mediated by transferring miR-410 to the LUAD cells to inhibit PTEN protein expression. Our findings provide novel insights into the intercellular communications between cancer cells and MSCs through MSC-EV-miRNA. This study indicates that manipulation of hUCMSC and/or their EVs might be a therapeutic option to reduce the side effects caused by hUCMSCs or hUCMSC-EVs in clinical applications.

## Materials and Methods

### Preparation of hUCMSCs

Fresh umbilical cords were obtained from informed, consenting mothers at the First People’s Hospital of Nanjing (China) and rapidly processed. The cords were rinsed twice with phosphate-buffered saline (PBS) supplemented with penicillin and streptomycin (pen/strep; Gibco, Carlsbad, CA), and the cord vessels were removed. The washed cords were subsequently cut into small fragments that were individually attached to the substrate of culture plates. This was followed by the addition of stem cell culture medium (Cyagen, Guangzhou, China) and incubation at 37 ℃ with 5% CO_2_. The medium was replaced every 3 days after the initial culture, and well-developed colonies of fibroblast-like cells appeared ~10 days later. The colonies were then trypsinized (Gibco) and passaged into new plastic plates for further expansion. The human umbilical cord MSCs (hUCMSCs) from passages 3–7 were used for all the experiments. The experimental protocol was approved by the Nanjing Medical University Ethics Committee.

The osteogenic, adipogenic, and chondrogenic differentiation identified by Alizarin Red staining, Oil Red O staining, and Alcian Blue staining, respectively. To accomplish the differentiation, passages 3–7 MSCs were cultured in OriCell™ hUCMSCs osteogenic, adipogenic, or chondrogenic differentiation media (all from Cyagen) as described by manufacturer.

After the third passage, the hUCMSCs were trypsinized and washed twice with PBS; they were then stained with human anti-CD105, anti-CD73, anti-CD90, anti-CD45, anti-CD34, anti-CD14, anti-CD19, or anti-HLA-DR. Identical concentrations of FITC- or PE-conjugated mouse IgG isotype antibodies were used as negative controls (all from BD Biosciences Pharmingen, San Jose, CA). At least 10,000 events were acquired on a FACSVerse instrument (BD Bioscience), and the results were analyzed using FlowJo software (Tree Star, Ashland, OR).

### Cell culture

The human LUAD cell lines H1299 and PC-9 were purchased from ATCC (Manassas, VA). Cells were not revalidated for this work. All of the cell lines were grown in RPMI 1640 medium (HyClone, South Logan, UT) supplemented with 10% fetal bovine serum (Gibco) and 1% pen/strep (Gibco). The cells were incubated under 5% CO_2_ and 37 ℃ conditions.

### Generation of hUCMSC extracellular vesicles

The hUCMSCs were cultured until ~80% confluent in culture dishes, then washed twice with PBS and re-incubated with serum-free culture medium at 37 ℃ with 5% CO_2_. After 24 h, the hUCMSCs culture supernatants were collected, centrifuged at 300×*g* for 10 min to eliminate cells and centrifuged at 2000×*g* for 20 min, followed by filtration through a 0.45-μm filter to remove cell debris. Then, the filtered supernatants were collected and subjected to ultracentrifugation (Beckman Coulter Optima L-100 XP ultracentrifuge) at 100,000×*g* for 90 min at 4 ℃. The pellets were gathered and washed in PBS, subjected to a second ultracentrifugation, and resuspended in PBS. The particle size distribution of the hUCMSC-EVs was measured using a Zetasizer Nano ZS (Malvern Instruments Ltd,, Worcestershire, UK) according to the operating instructions. The quantification of hUCMSC-EVs number was counted by nanoparticle trafficking analysis using NanoSight NS300 (Malvern Instruments Ltd.) according to the manufacturer’s manual. The hUCMSC-EVs aliquots were stored at -80 °C until required.

### Transmission electron microscopy

The purified hUCMSC-EVs were fixed with 4% paraformaldehyde (PFA) and 4% glutaraldehyde in 100 mM phosphate buffer (pH 7.4) at the ambient temperature. Then, fixed hUCMSC-EVs were dropped onto a carbon-coated copper grid and immersed in 2% phosphotungstic acid solution (pH 7.0) for 30 s. The grid was examined using a transmission electron microscope (JEM-1200EX; JEOL Ltd., Tokyo, Japan).

### HUCMSC-EVs uptake

H1299 cells were incubated in serum-free culture medium containing 3,3-Dioctadecyloxacarbocyanine perchlorate (DIO) (green) cell-labeling solution for 20 min at 37 °C and washed twice with PBS. The hUCMSC-EVs were labeled with CM-Dil (red), and the excess dye was removed by using ultracentrifugation at 100,000×*g* for 1 h, then washed twice. After the CM-Dil-labeled hUCMSC-EVs were incubated with the DIO-labeled cells for 2 h, the cells were fixed with 4% PFA, permeabilized with 0.5% Triton-X 100 and washed, and subsequently, the cell nuclei were stained using 4,6 diamidino-2-phenylindole (DAPI). All reagents were from Invitrogen (Carlsbad, CA). Images of hUCMSC-EVs uptake were obtained using a Nikon Eclipse Ti confocal laser scanning microscope.

After incubation (12 h) of the H1299 cells with the CM-Dil-labeled hUCMSC-EVs, the cells were detached and evaluated using a FACSVerse instrument (BD Bioscience), and the results were analyzed using FlowJo software (Tree Star).

### Animal studies

Four-week-old female BALB/c nude mice (Laboratory Animal Center of Nanjing Medical University, Nanjing, China) were housed under specific-pathogen-free conditions.

The mice were randomly divided into four groups, and all groups received subcutaneous injections of 0.2 mL PBS per mice containing H1299 cells alone (1.5 × 10^6^), H1299 cells (1.5 × 10^6^) mixed with hUCMSCs (1.5 × 10^6^), H1299 cells (1.5 × 10^6^) mixed with 6 × 10^9^ hUCMSC-EVs (200 μg) according to previous reports^52,53^, or hUCMSCs alone (1.5 × 10^6^). The tumor sizes were measured with calipers every 7 days.

To rule out the indirect effects of hUCMSC-EVs via other non-umor cells, such as endothelial cells and immune cells, 1.5 × 10^6^ H1299 cells were treated with serum-free culture medium, hUCMSC-EVs (3 × 10^9^ per mL), MSC/scramble-EVs (3 × 10^9^ per mL) or MSC/siR410-EVs (3 × 10^9^ per mL) in vitro for 12 h, completely washed by PBS to remove the EVs remaining outside of LUAD cells and then subcutaneously injected LUAD cells with EVs inside into nude mice. The tumor sizes were determined with calipers every 7 days.

The tumor volumes were calculated with the following formula: (length × width^2^)/2. The animal studies were approved by the Animal Ethics Committee of Nanjing Medical University.

### Immunohistochemistry

Briefly, the tumor tissue slides were incubated with an antibody to human Ki-67 (Abcam, Cambridge, MA), followed by an HPR-conjugated secondary antibody, and DAB was used as the substrate. The nuclei were counterstained with Harris’s hematoxylin. Cell apoptosis was assessed using a terminal TUNEL assay using an In Situ Cell Death Detection Kit (Roche, Basel, Switzerland) according to the manufacturer’s instructions.

### Cell viability assay

The cell viability was analyzed using the cell counting kit-8 (KeyGEN BioTECH, Nanjing, China). H1299 or PC-9 cells were seeded in 96-well plates (5 × 10^3^ cells per well) and cultured overnight. Then, the medium was replaced with 100 μL of serum-free medium in the absence or presence of various concentrations of the hUCMSC-EVs for 24 or 48 h. Then, 10 μL of CCK-8 was added to each well, and the cells were incubated for 1.5 h (PC-9) or 3 h (H1299). Finally, the absorbance of the cells in each well was measured at 450 nm using a microplate reader (Synergy HT, BioTek, Biotek Winooski, VT). Culture medium without cells was used as the blank control.

### Cell proliferation and apoptosis assay

H1299 (3 × 10^5^ cells) or PC-9 (5 × 10^5^ cells) were plated in serum-free medium in six-well plates and treated with hUCMSC-EVs, hUCMSC-scramble-EVs, or hUCMSC -siR410-EVs.

Cell proliferation was measured using an iClick™ EdU Andy Fluor 647 Flow Cytometry Assay Kit (Genecopoeia, Germantown, MD), according to the manufacturer’s instructions. The percentage of EdU^+^ cells was determined using a FACSCalibur flow cytometer (BD Biosciences).

Cell apoptosis was measured by staining with fluorescein isothiocyanate-labeled Annexin V (Annexin V-FITC) and 7-amino-actinomycin D (7-AAD) (BD Biosciences) according to the manufacturer’s instructions. The percentage of apoptotic cells was evaluated using a FACSCalibur flow cytometer (BD Biosciences).

At least 10,000 events were acquired, and the results were analyzed using FlowJo software (Tree Star).

### Cell transfection

The hUCMSCs and H1299 cells at 60% confluency were transfected with 100 nM miRNA or H1299 cells were transfected PTEN plasmid (1 μg) using Lipofectamine 2000 (Invitrogen, Carlsbad, CA) in Opti-MEM (Invitrogen) according to the manufacturer’s procedures. The synthetic miR-410 mimic, miR-410 inhibitor, mimic control, and inhibitor control were purchased from RiboBio (Guangzhou, China). PTEN overexpression plasmid and control vector plasmid were purchased from Genecopoeia.

HUCMSC-EVs were collected from hUCMSCs transfected with the miR-410 mimic (FAM, green) and then labeled with CM-Dil (red), washed, and incubated with H1299 cells in serum-free medium for 6 h. Next, the cells were fixed with 4% PFA, permeabilized, and washed; then, the cellular nuclei were stained using DAPI. The green fluorescence and the red fluorescence were detected using a Nikon Eclipse Ti confocal laser scanning microscope.

### RNA isolation and quantitative real-time PCR

RNA was extracted from the hUCMSC-EVs, cell preparations, and tumor tissues using a mirVana RNA isolation kit (Ambion, Austin, TX) according to the manufacturer’s protocol. All of the primers for real-time PCR were purchased from Genecopoeia. Real-time PCR was performed with All-in-one™ qPCR Mix (Genecopoeia) in a CFX96™ Real-Time system (Bio-Rad, Hercules, CA). The relative expression of miRNAs or mRNA was evaluated by the 2^−ΔΔCt^ method and normalized to U48 or GAPDH, respectively, based on our previous description^54^.

### Western blotting

The hUCMSC-EVs, hUCMSCs, H1299 transfected with miRNA with or without stimulation with hUCMSC-EVs, and tumor tissues were collected in RIPA buffer (Cell Signaling Technology Inc., Danvers, MA) containing PMSF (Beyotime, Nantong, China) and quantified using a BCA Protein Assay Kit (Beyotime). Equal amounts of proteins (50 μg) were electrophoresed in 10% sodium dodecyl SDS-PAGE and transferred onto polyvinylidene difluoride membranes (Bio-Rad), as previously described^54^. The antibodies to human CD63, calreticulin, PTEN, and β-actin, as well as HRP-linked anti-rabbit IgG, were all purchased from Abcam.

### Luciferase reporter assay

H1299 or PC-9 cells were co-transfected with 500 ng pmiR-RB-report-h-PTEN-3′UTR (wild type and mutant type) and 50 nM miR-410 mimics, 100 nM miR-410 inhibitor, and negative control (ribobio) using Lipofectamine 2000 (Invitrogen) according to the manufacturer’s manual. After 48 h, cells were lysed to Luc-Pair™ Duo-Luciferase Assay Kit 2.0 (GeneCopoeia), and luciferase activity was measured. Luciferase activity was normalized by Renilla/Firefly luciferase signal in H1299 or PC-9 cells.

### Statistical analysis

The statistical analyses were performed with GraphPad Prism (Version 5.0; La Jolla, CA). The data are expressed as the means ± SD. Statistical significance was determined using the Mann–Whitney test or one-way analysis of variance, and a *P* < 0.05 was considered statistically significant.

## Electronic supplementary material

Figure S1

Figure S2

Figure S3

Figure S4

Figure S5

Supplementary Figure Legends
