# Supplementary figures and images for: Human umbilical cord mesenchymal stem cell-derived extracellular vesicles promote lung adenocarcinoma growth by transferring miR-410
Source: Cell Death Dis. 2018 Feb 13;9(2):218. doi: 10.1038/s41419-018-0323-5 (PMC5833395; doi:10.1038/s41419-018-0323-5)

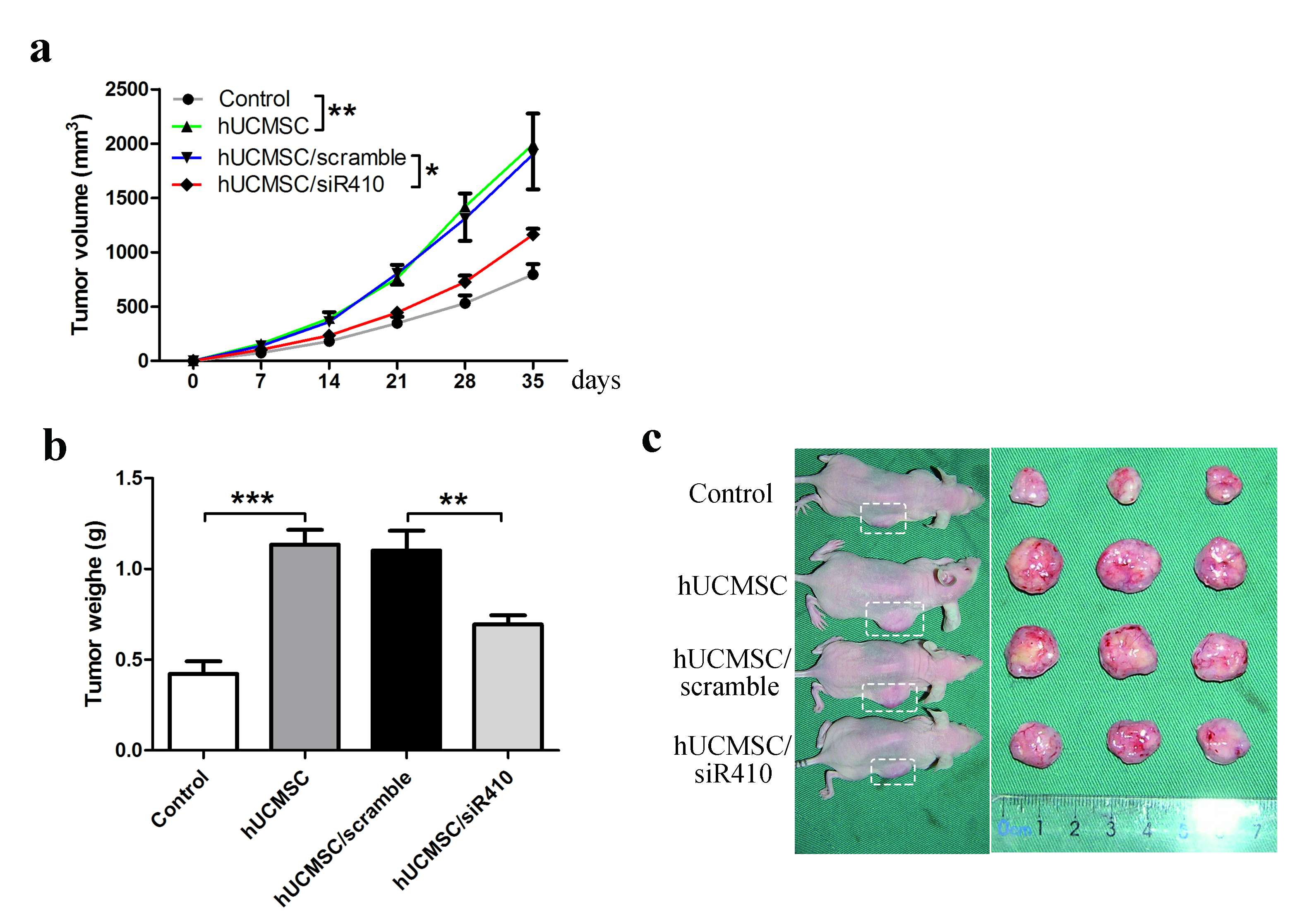

Supplement: Supplementary file 1 — Figure S1 [file 41419_2018_323_MOESM1_ESM.tif]

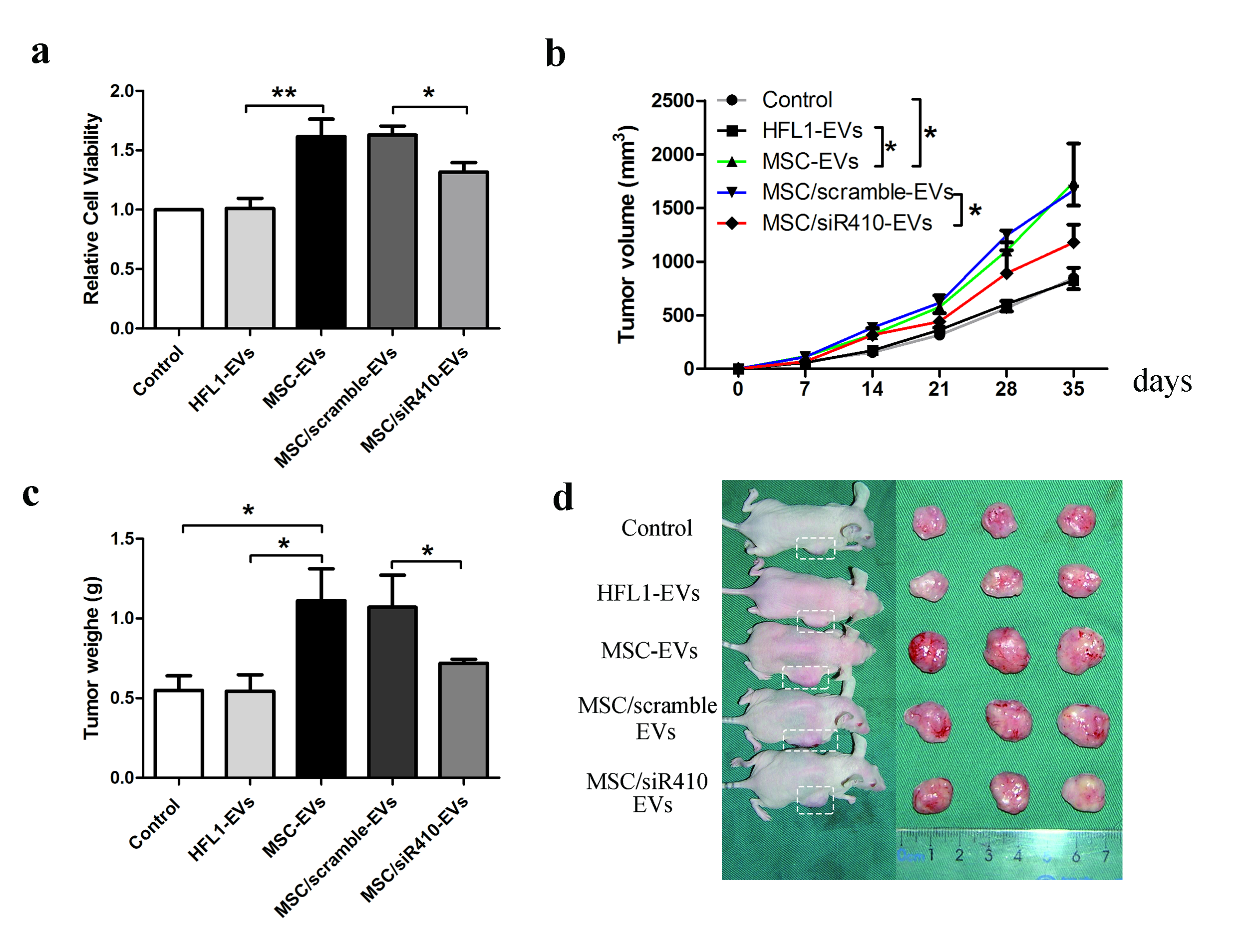

Supplement: Supplementary file 2 — Figure S2 [file 41419_2018_323_MOESM2_ESM.tif]

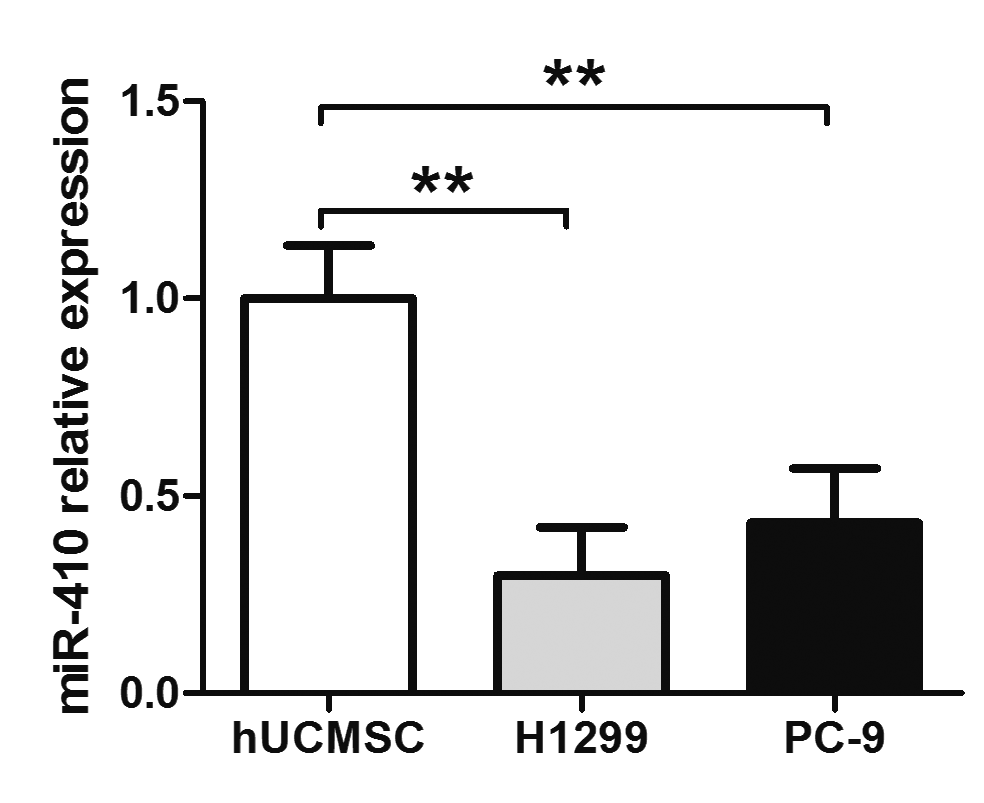

Supplement: Supplementary file 3 — Figure S3 [file 41419_2018_323_MOESM3_ESM.tif]

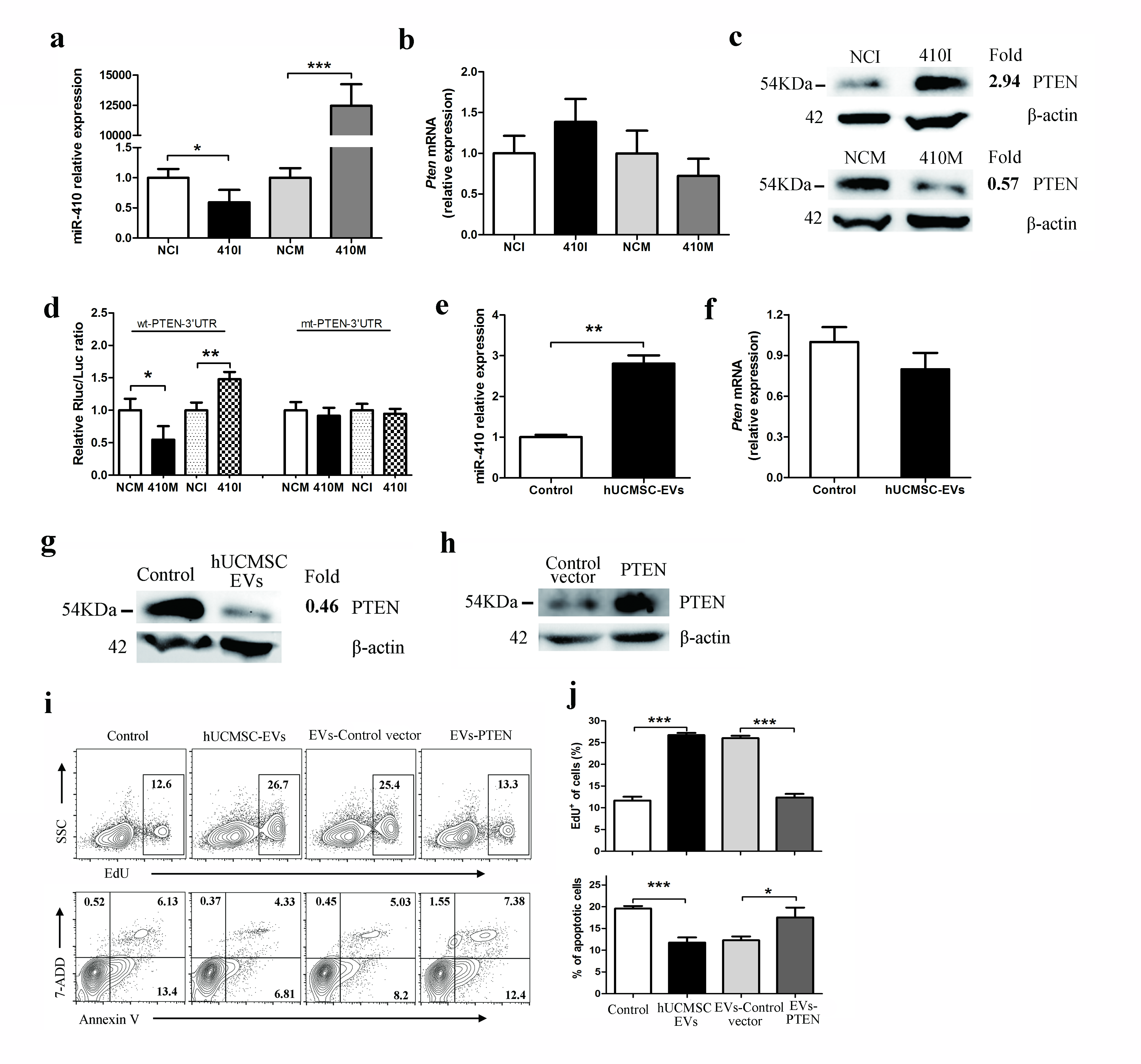

Supplement: Supplementary file 4 — Figure S4 [file 41419_2018_323_MOESM4_ESM.tif]

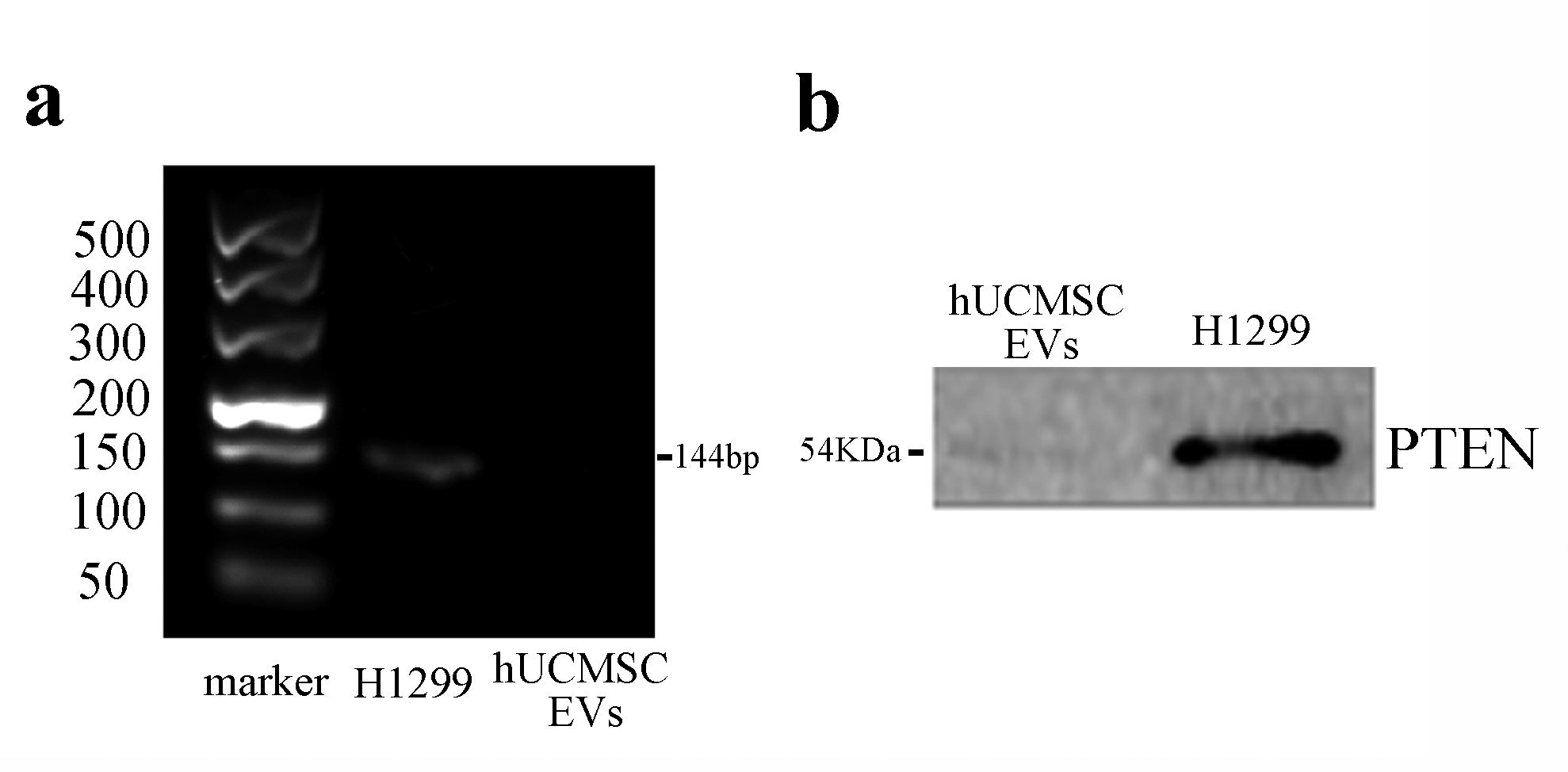

Supplement: Supplementary file 5 — Figure S5 [file 41419_2018_323_MOESM5_ESM.tif]
