# Supplementary material for: Human umbilical cord mesenchymal stem cell-derived extracellular vesicles promote lung adenocarcinoma growth by transferring miR-410
Source: Cell Death Dis. 2018 Feb 13;9(2):218. doi: 10.1038/s41419-018-0323-5 (PMC5833395; doi:10.1038/s41419-018-0323-5)
Supplement: Supplementary file 6 — Supplementary Figure Legends [file 41419_2018_323_MOESM6_ESM.docx]

**Supplementary Figure Legends**

**Figure S1** MiR-410 contributed to the promotion effect of hUCMSC on LUAD cell growth. (**a**) The tumor volumes in mice implanted with PC-9 cells alone (1.5×10^6^) or PC-9 cells (1.5×10^6^) co-implanted with hUCMSCs (1.5×10^6^), MSC/scramble (1.5×10^6^), MSC/siR410 (1.5×10^6^). (**b**) The tumor weights of the PC-9 xenograft tumors at 35 days post-implantation. (**c**) Imaging of the tumors and tumor-bearing mice at 35 days post-implantation.

**Figure S2** MiR-410 was involved in the hUCSMC-EVs-mediated LUAD cell growth *in vivo*. (**a**) PC-9 cells in serum-free media were incubated with EVs (1.5×10^9^/mL) for 48 h, and the cell viability was determined using the CCK8 assay. (**b-d**) The PC-9 cells (1.5×10^6^) were pre-stimulated with EVs (1.5×10^9^/mL) for 12 h before they were injected into the nude mice. (**b**) The tumor volumes were measured, and (**c**) the tumor weights were determined at 35 days. (**d**) Imaging of tumors and tumor-bearing mice at 35 days after the injections.

**Figure S3** The miR-410 expression was higher in hUCMSCs than that in LUAD cells. The expression of miR-410 was determined by real-time PCR in hUCMSCs, H1299 cells, or PC-9 cells.

**Figure S4** PTEN was a direct target of miR-410 in LUAD cells. (**a**) Real-time PCR was used to investigate the miR-410 transfection efficiency. (**b**) Real-time PCR analysis of the expression of *PTEN* mRNA in PC-9 cells at 24 h post-transfection of the miR-410 inhibitor or miR-410 mimics compared to their negative controls. (**c**) PTEN protein expression was analyzed by western blotting at 48 h post-transfection with β-actin as a loading control. The intensity of each band was analyzed using ImageJ software. The ratios of the miR-410 inhibitor to the inhibitor control and of the miR-410 mimics to the mimic control are presented as the fold differences. (**d**) MiR-410 suppressed the luciferase activity that carried wild-type but not mutant 3’UTR of PTEN. (**e-f)** PC-9 cells were treated with hUCMSC-EVs (1.5×10^9^/mL) for 24 h or 48 h. (**e**) The expression of miR-410, (**f**) *PTEN* mRNA, and (**g**) PTEN protein was determined by real-time PCR or western blotting, respectively. (**h**) PC-9 were transfected with control vector plasmid or PTEN overexpression plasmid and the PTEN protein expression were analyzed by western blot. (**i, j**) PC-9 cells, which were transfected with PTEN overexpression plasmid, were incubated with/without hUCMSC-EVs (1.5×10^9^/mL) for 24 h or 48 h. (**i**) Representative flow cytometric contour plots of the EdU-stained PC-9 cells (24 h) and the Annexin V/7-ADD dual-stained PC-9 cells (48) were shown. (**j**) The percentages of EdU-positive cancer cells and both early and late apoptotic cells were shown.

**Figure S5.** The expression of *PTEN* mRNA and protein in hUCMSC-EVs. (**a**) Gel electrophoresis of *PTEN* mRNA in hUCMSC-EVs. (**b**) Western blotting analysis of PTEN protein expression in hUCMSC-EVs. The protein expression of PTEN in H1299 cells was used as a positive control.
